# UHPLC-MS/MS Analysis of the Accumulation and Excretion of Steroidal Glycoalkaloids Consumed by Potato Tuber Moth (*Phthorimaea operculella*) Larvae under Different Feeding Treatments

**DOI:** 10.3390/insects14010026

**Published:** 2022-12-26

**Authors:** Yajin Li, Qiong Wang, Xiaoyu Xu, Huachun Guo

**Affiliations:** 1 College of Agronomy and Biotechnology, Yunnan Agricultural University, Kunming 650201, China; 2 Plant Protection College, Yunnan Agricultural University, Kunming 650201, China; 3 Tuber and Root Crops Research Institute, Kunming 650201, China

**Keywords:** SGA, UHPLC, *Phthorimaea operculella*

## Abstract

**Simple Summary:**

Food poisoning caused by potato glycoside alkaloids (SGA) remains a critical factor affecting potato production safety. The potato tuber moth is a notorious pest that damages all parts of potato tissues. In this study, the dominant SGA substances α-solanine and α-chaconine in different potato leaves were detected. The growth and development of PTM feeding on the leaves of different potato varieties and different SGAs containing an artificial diet were studied. To clarify the SGA concentration accumulation and excretion processes, the tissues of potato tuber moth larvae were dissected into several parts and studied using an UHPLC machine. The results showed that ecdysis and the excretion process may be effective approaches used by the potato tuber moth to equilibrate internal SGA accumulation.

**Abstract:**

Food poisoning caused by potato glycoside alkaloids (SGA) remains a critical factor that affects potato production safety. The potato tuber moth (*Phthorimaea operculella*) is a notorious pest that displays good adaptability to SGA in potato tissues. Studies that explore the mechanisms underlying SGA homeostasis in potato tuber moth larvae are urgently needed. In this study, ultra-high-performance liquid chromatography (UHPLC)-triple quadrupole mass spectrometry (MS/MS) was applied to detect the dominant SGA substances α-solanine and α-chaconine in potato leaves and PTM larval tissues. From the highest to lowest SGA concentrations, the potato cultivars studied were ranked as follows: DS47, LS6, DS23 and QS9. To exclude the influence of nutrients within different potato varieties, different SGA containing (0%, 0.1%, 0.2%, 0.3% and 0.4%) the artificial diet treatment groups were added. DS47 and 0.3% SGA-containing artificial diets presented the best conditions for PTM growth, development and reproduction compared to other potato cultivars and artificial diet controls. The potato tuber moth larva tissues were dissected and the SGA content within different tissues were detected using an UHPLC machine. The results showed that α-chaconine was dispersed in the feces, midgut, hindgut, head and cuticle, and α-solanine was distributed only in the feces and midgut. Antibiotic-treated insects exhibited higher concentrations of SGA than the normal microbiome group. Furthermore, the SGA concentrations of 100 newly-hatched PTM larvae and puparia were detected, with both of them found to contain small amounts of SGA. The results showed that ecdysis and the excretion process were effective approaches used by the potato tuber moth to equilibrate internal SGA accumulation. The microorganism-decreased SGA concentrations were excited in their gut. SGA may transfer from adults to the next generation, and SGAs in PTM are inheritable. In this study, we demonstrated that the potato tuber moth possessed an effective method to preliminarily decrease high SGA accumulation in potato.

## 1. Introduction

The potato tuber moth *Phthorimaea operculella* (Lepidoptera: Gelechiidae), a soft-bodied insect with chewing mouthparts, is a unique, potato-specific pest that can feed on all parts of the potato plant, despite the plant’s ubiquitous steroidal glycoalkaloid (SGA) accumulation. This phenomenon has not yet been found in the other 50 potato pest species [1]. Potato tuber moth (PTM) larvae have four instars and have excellent fecundity. They spend their whole life cycle on SGA-rich potato tissues, mining the leaf, stem and petioles and excavating tunnels into the tuber to feed. Therefore, pest control of PTM has become rather difficult [2,3,4,5]. Currently, PTM occurs in nearly all tropical and subtropical potato-growing regions in Africa and Asia, as well as in North, Central and South America, making it the most widely-spread potato pest in the world [4,6].

Potato (*Solanum tuberosum*) is the fourth-largest staple food crop in the world [7,8]. Unlike other staple crops, such as rice (*Oryza sativa*), maize (*Zea mays*) and wheat (*Triticum aestivum*), the enrichment of SGAs in potato tissues (which are a group of secondary plant metabolites toxic to insects, animals and humans) poses a potential threat to potato production and industrial development [9,10]. Presently, more than 100 types of SGAs have been identified in potato, and the main components, α-solanine (C_45_H_73_O_15_N) and α-chaconine (C_45_H_73_O_14_N), account for more than 95% of the total SGAs [11,12,13,14,15]. Generally, the potato fruit contains the highest level of SGAs (1.0%), followed by the flowers (0.73%), buds (0.73%), leaves (0.25%) and tubers (0.004%) [9,16]. Potato’s glycoalkaloid content may increase dramatically during storage or transportation, or may even be induced by environmental and physiological factors, including light, heat, wounding, sprouting and pest invasion [9]. Glycoalkaloid poisoning might be exacerbated when the SGA content exceeds 20 mg/100 g FW [17]. The SGA content in potato mainly depends on genotype differences and environmental factors, such as soil, light exposure and shade [18,19].

During long-term evolution, many plants gradually formed a complex defensive system to resist a/biotic stresses [20]. For example, toxic secondary metabolites generated under adverse circumstances have the ability to affect the feeding, digestion, growth and development of pests, sometimes even herbivores. Phenylpropanoids and limonoids have been shown to deter oviposition in the naive moths of *Trichoplusia ni* in choice tests [21]. A diet of *T. ni* and *Depressaria pastinacella* containing 1% phytic acid caused 100% mortality for first instar larvae after 120 h [22]. Further, the mortality of *Spodoptera frugierda* increased significantly when 1% rotenone was added to their diet [23]. To overcome such barriers preventing food intake, strategies against plant chemical defenses have also been developed by insect herbivores, among which diet breadth has become the most applicable [24]. Particularly, specialist herbivores are able to tolerate the defensive metabolites of their host plants or even benefit from them for their own development or as anti-predatory defenses [25,26,27]. Potato plants stressed by Colorado potato beetles (*Leptinotarsa decemlineata*) produce tubers containing higher α-chaconine and α-solanine concentrations [9]. However, a study found that a higher α-chaconine level negatively correlated with PTM larval mortality. In other words, the more α-chaconine produced, the less PTM resistance a potato plant had [28]. Although a number of studies have reported that SGAs are resistant to multiple phytophagous insects, the current research on potato pests is still focused on control, resistance and pesticide toxicology [2,29,30,31,32,33]. The methods by which PTM larvae survive and complete their full life cycle on potato plants equipped with natural insecticide SGAs remain to be investigated.

Normally, exuviation and feces are the main routes herbivorous insects use to attenuate in vivo plant secondary metabolite repercussions via release through excretion [18]. Self et al. [34] detected four alkaloids corresponding to tobacco plants in *Trichoplusia ni*‘s feces after feeding the insect nicotine-containing feed for a certain period of time. This is an effective method to prevent potential toxins from passing the insect’s gut wall and rendering target sites inaccessible. An insect typically stores food in its foregut, with its midgut undertaking the subsequent digestion and absorption of nutrients and the catalysis of toxic substances [35]. Hence, it was important to explore whether PTM harbored similar physiological mechanisms in its defense against SGAs derived from potato plants. In this study, an UHPLC–MS/MS system was utilized to determine the contents of α-solanine and α-chaconine in fourth instar PTM larvae after feeding them various potato tissues. Several tissues of the PTM larvae, including the head, cuticula, foregut, midgut and hindgut, were analyzed in order to determine the metabolic dynamics of SGAs within the insect. PTM has long posed a threat to global potato production, while low SGA potato germplasms are increasingly needed by the market to better fit current food processing requirements [36,37]. As a consequence, the strategic development of both potato breeding and pest control should be simultaneously considered. Our results could provide preliminary evidence to better understand the interactions between potato plants and the PTM, as well as SGA homeostasis in PTM larvae.

## 2. Materials and Methods

### 2.1. Plant Materials

Four potato cultivars of tubers, namely Dianshu 47 (DS47), Dianshu 23 (DS23), Qingshu 9 (QS9) and Lishu6 (LS6), were obtained from the Tuber and Root Crops Research Institute, Yunnan Agricultural University. These potato cultivars are widely cultivated crops with high yields in Yunnan Province. Potato cultivars were planted in pots and kept in the greenhouse of Yunnan Agricultural University, P. R. China, with no pesticides and no other pests involved during the growth period. All the potato cultivars were planted on the same date except DS47 (with low germination), and 30 plants of each were planted (3 plants in one pot). The same genotype was planted every week until the larvae pupated, and the leaves were fed after they were picked to ensure that the larvae had access to fresh and tender leaves. When the plant grew to 30 cm, the middle leaves were cut to allow PTM larvae to feed on them. Then, the third or fourth potato leaves were collected. Three replicates per variety were weighed and then frozen in liquid nitrogen for SGA content analysis.

### 2.2. Insect Rearing Method

*Phthorimaea operculella* larvae were collected from a potato field in Tuanjie Town, Kunming City, Yunnan Province, and maintained in an insectary without exposure to any insecticide. The population of potato tuber moths was reared on QS9 tubers in insect rearing cages at 25 ± 2 °C and 70 ± 5% relative humidity with a photoperiod of 16:8 h (L:D). Adults were supplemented with a 10% honey solution under the same conditions [38]. After the adults were mated, they were placed in a petri dish and covered with a double layer of gauze with 10% honey to collect their eggs. Twelve hours later, gauze was taken and the eggs were used for insect rearing experiments.

#### 2.2.1. Potato Tuber Moth Feeding on Potato Leaves

The larvae of the potato varieties treatment group were reared on the leaves of different potato varieties (DS47, DS23, LS6, QS9) in their growth and development period. Insect rearing was continued for two generations on each cultivar before the insects were used in the experiments. A total of 3 replicates per variety and 20 individuals were randomly selected to have their body weights, body lengths, and head capsules measured every day, as well as to record the larva stage feeding on different potato varieties’ leaves. The pupa stage was counted and the reproduction parameters were collected after adults emerged and mated (one female to one male, five replicates) and eggs hatched. All data were presented as the mean ± standard error.

#### 2.2.2. Potato Tuber Moth Feeding on Artificial Diet

The different SGA group, which contained the artificial diet treatment group, was reared under the same conditions as the potato leaf group. Standard solutions of α-solanine (400 μg mL^−1^) and α-chaconine (200 μg mL^−1^) were prepared by making a stock solution in methanol, which was stored at −20 °C until use. The insect artificial diets were from Kashyap et.al. [39]. Five grades were set with 100 mL of each: 0%, 0.1% (0.5 mL of α-chaconine:0.25 mL of α-solanine), 0.2% (1 mL of α-chaconine:0.5 mL of α-solanine), 0.3% (1 mL of α-chaconine and 0.75 mL of α-solanine) and 0.4% (2 mL of α-chaconine:1 mL of α-solanine). An artificial diet without SGA content was used for the control group (0%). They were reared on DS47 leaves in the first and second instars, as this is the best time for PTM larvae to be consumed. Then, the 3rd freshly-molted larvae were transferred to the artificial diet. The growth and reproduction parameters were recorded in order to select the best conditions for PTM larva growth under different food treatments.

#### 2.2.3. Potato Tuber Moth Feeding on Antibiotic Artificial Diet

To test the hypothesis that SGA degradation is a microbially-mediated PTM process, we designed the experiment. Measurements of SGA concentrations by UHPLC–MS/MS were carried out using the same method as above. To determine involvement of the microbiota in SGA degradation, we eliminated microorganisms using the defined diet, supplemented with two broad-spectrum antibiotics, tetracycline and streptomycin (Shanghai Yuanye Biological Technology Co., Ltd., Shanghai, China). First, a PTM laboratory-reared colony with the same conditions was grown to the third instar and then transferred to an artificial diet (containing 0.3% SGA) for 36 h (Control) in a controlled experiment using DS47 leaves. Second, the third PTM larvae in the first step were fed an artificial diet containing antibiotics (0.025 mg mL^−1^) after hunger treatment for 6 h. Third, one part of larvae in the second step were fed an artificial diet containing SGA for 36 h again. Then, larval midgut tissues from all three steps above were dissected for further SGA analysis.

### 2.3. Sample Preparation

For the quantitation of glycoalkaloids (α-chaconine and α-solanine) in the potato tuber moth, exuviae and feces of larvae from two selected treatments with different foods were weighed in a 5-mL memory tube. The tissues of potato tuber moths, including the head, cuticula, foregut, midgut and hindgut, were weighed after dissection. Due to the small body size of potato tuber moth larvae, only the fourth instar larvae were used in this study. The larvae in good condition from Section 2.2.2 and Section 2.2.3 were selected for dissection, with three replicates of each carried out. 

Samples were ground completely and then extracted with 2 mL of 5% aqueous acetic acid (5:95, *v*/*v*), with overnight maceration in the laboratory at room temperature (25 °C). The material tube was centrifuged for 10 min in a high-speed refrigerated centrifuge at 4 °C and 8000 r/min. Then, 1 mL of supernatant was drawn and diluted with 80% acetonitrile solution to a volume of 5 mL. A 2.5-mL syringe was used to aspirate the solution through a 0.22-µm organic filter, and the solution was transferred to a 1.5-mL HPLC sample bottle for UHPLC–MS/MS detection. A 0.22-µm nylon syringe filter was obtained from Biosharp (BS-QT-013, Anhui, China). The material extracts feeding on the artificial diet were chosen as the quality control sample.

Standard solutions of α-solanine (400 μg mL^−1^) and α-chaconine (200 μg mL^−1^) were prepared by making a stock solution in methanol and storing it at −20 °C. Working standard solutions were prepared by dilution of the stock solution in methanol.

### 2.4. Ultrahigh-Performance Liquid Chromatography-Triple Quadrupole Mass Spectrometry Analysis

The determination of PTM larva glycoalkaloid content followed a method previously used for potato tissues [13,40]. Ultra-high-performance liquid chromatography-tandem mass spectrometry (UHPLC-MS/MS) (Thermo Fisher Scientific, Waltham, MA, USA) and Hypersil Gold Syncronis C18 (100 × 2.1 × 1.7 µm, Thermo Fisher Scientific, Bellefonte, PA, USA) columns were utilized. The mobile phase consisted of 0.1% purified water and 0.1% aqueous formic acid as solvent A and 100% methanol as solvent B. The gradient started at 10% B, increased to 100% B over 3 min and was held at 100% B for 2 min before returning to 10% B over 1 min for column washing and stabilization. The flow rate was set to 0.35 mL/min, the injection volume of the samples and standards was 5 μL and the column temperature was 35 °C.

The heated electrospray ionization (ESI) source (H-ESI, Thermo Fisher Scientific, USA) was operated in positive ion mode. The source parameters were as follows: spray voltage, static; positive ion, +3.0 kV; negative ion, +3.0 kV; sheath gas (N_2_) flow rate, 40 (arbitrary units); auxiliary gas (N_2_) flow rate, 10 (arbitrary units); sweep gas flow rate, 0 (arbitrary units); ion transfer tube temperature, 330 °C; vaporizer temperature, 330 °C. The orbitrap spectrometer was operated with a resolution of 35,000 and a mass range of *m*/*z* 300–1000. Data processing was performed using Thermo Xcalibur Quan Browser software (Version 4.1, Thermo Fisher Scientific, USA). Concentrations of α-chaconine and α-solanine in samples were quantified using external calibration curves made from commercial standards of both α-chaconine and α-solanine (α-chaconine CAS NO. 20562-03-2; α-solanine CAS NO. 20562-02-1, Extrasynthese, France). A high linear correlation between SGA concentration and ion intensity (R^2^ = 0.9997 for α-chaconine and R^2^ = 0.9996 for α-solanine) was obtained at 1 to 400 ng L^−1^. Chromatographic grade methanol, acetonitrile, formic acid and ammonium acetate were purchased from Sigma (Saint Louis, MO, USA). Wahaha purified water (Wahaha Group, Zhejiang, China) was used as purified water.

### 2.5. Data Analysis

Total SGA were expressed as the sum of α-chaconine and α-solanine [40]. The data were analyzed by *t*-test and variance analysis. Before the analysis, homogeneity of variance and normality tests were conducted. If the criteria were not met, logarithmic conversion was performed on the data. In the process of ANOVA, if significant differences were detected, multiple comparisons were made using Duncan’s method. The data analysis software used was SAS v.9.2 (SAS Institute Inc., Cary, NC, USA). The significance level was 0.05, and data were expressed as the mean ± SEM throughout.

## 3. Results

### 3.1. Effects of Different Potato Varieties on the Growth and Reproduction of Potato Tuber Moths

#### 3.1.1. SGA Concentrations of Different Potato Varieties

**Figure 1:**
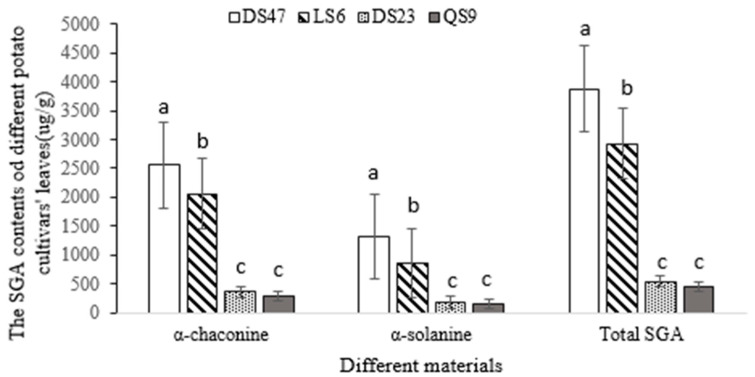
SGA concentrations in leaves of different potato cultivars. Different letters upon the column diagram are significantly different (*p* < 0.05, Tukey’s test).

The total SGA concentration in the DS47 potato leaf tissue was higher than that of the other varieties, recorded as 3879.72 ± 65.37 µg/g (α-chaconine, 2639.41 ± 300.36 µg/g and α-solanine, 1311.73 ± 83.98 µg/g). QS9 recorded the lowest total SGA content of 457.02 ± 38.82 µg/g (α-chaconine, 296.90 ± 321.72 µg/g and α-solanine, 185.67 ± 13.38 µg/g) of leaf biomass (flesh weight) (n = 3) (F = 11.78; df = 3; *p* < 0.05). LS6′s SGA concentration (α-chaconine, 2056.13 ± 210.44 µg/g; α-solanine, 863.52 ± 41.34 µg/g) was slightly lower than that of DS47. The SGA contents in the potato leaves of DS23 and QS9 showed no significant differences. From the highest to lowest SGA concentrations, the varieties were ranked as follows: DS47, LS6, DS23 and QS9 (Figure 1), with the same ranking occurring for the contents of α-chaconine and α-solanine. Otherwise, α-chaconine concentrations were higher than α-solanine concentrations at all potato genotypes leaves (Figure 1).

#### 3.1.2. Effects of Different Potato Varieties on the Growth and Reproduction of Potato Tuber Moths

The growth and development of PTM feeding on the leaves of different potato varieties were studied. It was noted that higher SGA content correlated with longer development time of the larvae. The DS47 feeding treatment group had the longest development time (16 d); the next longest was LS6 (14.5 d), and the QS9 and DS23 feeding groups both had a development time of approximately 12 d. The body lengths and head widths of the four treatments were not different, but the differences in body weights were significant, with the DS47 group having the largest body weights in the fourth instar (8.10 ± 1.51 mg /individual) and the QS9 group having the lowest (4.48 ± 1.60 mg/individual) (Table 1).

The results of the reproduction parameters of PTM feeding on potato leaves are presented in Table 2. The pupal stage differed among larvae feeding on different potato cultivars; it was longest for larvae feeding on DS47 (8.0 ± 0.63 d) and shortest for those feeding on DS23 (5.5 ± 1.30 d). The larvae feeding on DS47 had the highest pupal weights (9.47 ± 1.39 mg), and those feeding on QS9 recorded the lowest pupal weights (7.07 ± 1.40 mg). The pupal rate varied from 68% on QS9 to 84% on DS23, LS6 and DS47. All the emergence rates were above 80%, with the larvae feeding on DS47 recording the highest emergence rate (92%). The potato cultivars significantly affected the pupal weight. From highest to lowest, the fecundity rates obtained from the potato cultivars were ranked as follows: DS47, LS6, DS23 and QS9. The mean number of eggs laid per female was affected significantly by the potato cultivars; the highest number was recorded on DS47 (89.5 ± 60.28 eggs) and the lowest on QS9 (57 ± 36.35 eggs). The highest and lowest egg hatchability values were observed on DS47 (76.7 ± 6.32 eggs) and QS9 (41 ± 3.11 eggs), respectively (Table 2). DS47 presented the best conditions for PTM growth, development and reproduction.

### 3.2. Effects of Different SGAs Containing an Artificial Diet on the Growth and Reproduction of Potato Tuber Moths

The results of the reproduction parameters of PTM feeding on different SGA containing an artificial diet are presented in Table 3. The time from the larval stage to the pupal stage was affected significantly by the SGA concentration; it was longest in the 0.4% treatment (15.8 ± 1.46 d). The higher the SGA concentration was, the longer the survival time of the PTM larvae, with the pupation time being delayed at the same time. The pupal rate varied from 40.21% on 0.4% SGA to 72.92% on 0%, 0.1%, 0.3% on 0.2% SGA. Both the 0.2% and 0.3% SGA treatments had emergence rates over 90%, while the 0.3% SGA (95.45%) treatment had the highest emergence rate among all the treatments. The mean number of eggs laid per female was affected significantly by the SGA concentrations; it was the highest at 0.3% (70 ± 10.21 eggs) and lowest at 0.4% (29 ± 1.39 eggs). The highest and lowest egg hatchability values were observed for the 0.3% SGA (57.01 ± 3.39 eggs) and 0.4% SGA treatments (8.79 ± 1.23 eggs), respectively (Table 3).

### 3.3. SGA Accumulation and Excretion in Potato Tuber Moth Larvae

#### 3.3.1. Excretion and Accumulation of SGA in Potato Tuber Moth Larvae Feeding on Potato Leaves

**Figure 2:**
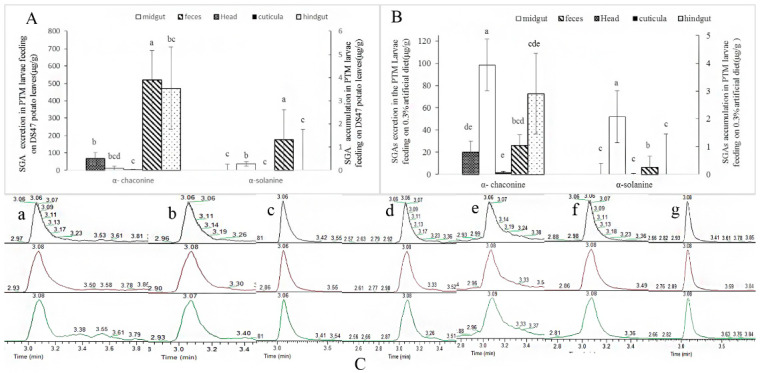
SGA accumulation and excretion in potato tuber moth larvae feeding on 0.3% artificial diet and potato leaves ((**A**), artificial diet feeding; (**B**), DS47 leaf feeding; (**C**), total ion current (TIC) and extract ion (EI) profiles at 852.70 *m*/*z* ([M + H]^+^ ion for α-chaconine) and 868.70 *m*/*z* ([M + H]^+^ ion for α-solanine) of the *Physorimaea operculella* samples: (a) foregut; (b) midgut; (c) hindgut; (d) head; (e) cuticula; (f) feces and exuvia; (g) exuvia).

SGA excretion and accumulation were detected in the PTM larvae feeding on DS47 leaves, which were the larvae characterized by the best developmental conditions (Table 1 and Table 2). α-chaconine and α-solanine were not detected in the foregut of the PTM larvae, and the excretion and accumulation of α-chaconine were higher than those of α-solanine (Figure 2A). α-chaconine was detected in the head, midgut, hindgut, cuticula and feces, and α-solanine was detected in the midgut (36.60 ± 0.04 μg/g) and feces (177.16 ± 1.61 μg/g) (*p* < 0.01). Otherwise, high levels of α-chaconine and α-solanine were detected in the feces (α-chaconine 519.42 ± 3.46 μg/g, α-solanine 177.16 ± 1.61 μg/g) and, from the highest to lowest α-chaconine concentration accumulation, the tissues of PTM were ranked as follows: the midgut (10.40 ± 0.02 μg/g), hindgut (3.54 ± 0.05 μg/g), head (0.51 ± 0.01 μg/g) and cuticle (0.03 ± 0.00 μg/g). The head and cuticle were characterized by low but significant α-chaconine accumulation (t = 12.35, *p* < 0.01). Excreting SGA through their feces is the most direct and effective method that potato tuber moths use to degrade SGA.

#### 3.3.2. SGA Accumulation and Excretion in PTM Larvae Feeding on Artificial Diet

Next, SGA excretion and accumulation in the PTM larvae feeding on the 0.3% SGA-containing artificial diet were detected, because these larvae had the best growth and developmental conditions (Figure 2B). α-chaconine concentrations in the midgut were higher (98.36 ± 6.93 μg/g) than those in the feces (26.02 ± 0.11 μg/g) (*t* = 17.96, *p* < 0.01); meanwhile, the head (0.80 ± 0.14 μg/g) and cuticula (0.07 ± 0.03 μg/g) recorded lower α-chaconine concentrations than both of these (*t* = 1.56, *p* > 0.05). α-solanine was detected only in the feces and midgut of the larvae feeding on this artificial diet, while the α-solanine content in the midgut (51.89 ± 19.61 μg/g) was higher than that in the feces (6.47 ± 0.13 μg/g) (*t* = 3.09, *p* < 0.05). Neither α-chaconine nor α-solanine were detected in the foregut of these larvae. α-chaconine accumulated in the cuticula, hindgut, midgut and head of the larvae, whereas α-solanine only accumulated in the midgut and feces. In addition, the SGA peaks in different tissues of these larvae are presented in Figure 2C(a–f); together with the exuviae of the larvae (Figure 2C(g)), a certain amount of α-chaconine and α-solanine was qualitatively analyzed herein, showing that exuviae were another method that larvae used to decrease SGA concentrations.

### 3.4. Detoxification of SGAs in the Midgut of Potato Tuber Moth Larvae

Antibiotic-treated insects exhibited higher concentrations of SGA, with concentrations in their gut being lower than those in the diet group. Furthermore, after being reinfected with SGA, α-chaconine and α-solanine concentrations were decreased (Table 4). It was speculated that the gut microbiota played a role in SGA degradation, although this would need to be further clarified.

### 3.5. The SGA Heredity of Potato Tuber Moth

**Figure 3:**
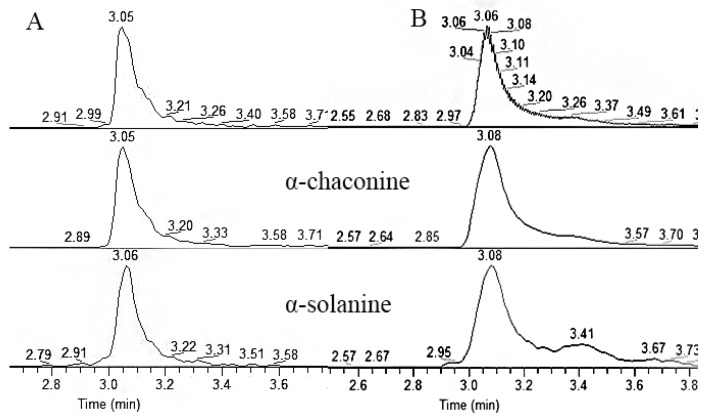
SGA concentrations in PTM tissue ((**A**), newly-hatched PTM larvae; (**B**), puparium.).

The SGA concentrations of 100 newly-hatched PTM larvae and puparia were detected for parental heredity identification. The results showed that the concentration of α-chaconine was 0.008 ± 0.002 μg/g, while α-solanine was not detected in the first larval stage (Figure 3A). The concentrations of SGA in puparia were detected qualitatively because of their low weights and were found to contain small amounts of SGA (Figure 3B). It could therefore be speculated that SGA transfers from adults to larvae.

## 4. Conclusions

From the highest to lowest SGA concentrations, the potato varieties studied were ranked as follows: DS47, LS6, DS23 and QS9. The α-chaconine concentration in the potato leaves was higher than the α-solanine concentration. The larvae feeding on DS47 leaves and the 0.3% SGA-containing artificial diet presented the best conditions for PTM growth, development and reproduction. The larvae preferred to feed on high SGA-containing food—i.e., the higher the SGA concentration, the longer the survival time of the larvae—with their pupation time being simultaneously delayed.

Exuviae and feces are effective methods used by PTM larvae to decrease their internal SGA concentrations. α-chaconine was found to have accumulated in the tissues of the larvae, such as in the cuticula, hindgut and head, whereas α-solanine accumulated only in the midgut. Neither α-chaconine nor α-solanine accumulated in the foregut of the larvae. Antibiotic-treated insects exhibited higher concentrations of SGA, showing that some intestinal microorganisms could degrade SGA in the midgut of PTM larvae. Furthermore, the SGA substances in newly-hatched PTM larvae and puparia were detected, showing that SGA could be transferred from adults to larvae. Further studies on SGA degradation and interactions with potato tuber moth mechanisms will be needed for PTM management.

## 5. Discussion

In this study, the SGA concentrations of four potato leaves (DS47, DS23, LS6 and QS9) from four potato varieties during the growing period were detected, with the results showing that the different varieties had different SGA contents. In order to clarify SGA concentrations’ effect on potato tuber moth growth and development, the SGA contents of the different varieties were detected again before feeding, and the results were in accordance with Li et. al. [41]. Genotypic variation was found to be the main factor influencing SGA accumulation in potato plants [19]; season and soil effects were not considered in this study. The growth and development parameters of larvae feeding on the artificial diet containing different concentrations of SGA were not measured, because young PTM larvae cannot survive by feeding on this diet. Alipour and Mehrkhou [42] studied the effects of six potato cultivars (Agria, Florida, Impala, Picasso, Satina, and Sprint) on the life history, life table and demographic parameters of *P. operculella* under laboratory conditions, but they did not study the potato cultivars’ SGA contents. Toxic secondary metabolites have the ability to affect insect feeding, digestion, growth and development. In this study, PTM larvae with good conditions were fed on the higher SGA-containing potato cultivar DS47 and the 0.3% SGA-containing artificial diet; the adaptive mechanism behind this calls for further study. Pacifico et al. [28] found that a higher α-chaconine level was negatively correlated with PTM larval mortality; in other words, the more α-chaconine produced, the lower the PTM resistance. In our study, the results for larvae fed on the higher SGA-containing artificial diet with the aforementioned production parameters were consistent with those of a previous study (Table 3). Both the potato leaf and artificial diet feeding showed that higher SGA concentrations delayed the PTM larval development time. Rainio et al. [43] stated that the long-term upregulation of antioxidant enzyme activity was energetically costly and could increase oxidative stress in these larvae, in turn delaying their developmental time.

Exuviae and feces are two of the main methods herbivorous insects use to resist plant secondary metabolites [18]. In this study, PTM feces were found to have high SGA concentrations, demonstrating that potato tuber moths were able to directly and effectively reduce toxic SGA in the body through excretion. The exuviae of the larvae analyzed were difficult to separate from the feces when the larvae were feeding on the potato leaves; when they were fed on the artificial diet, the exuviae separated well from the feces, but they were too light to weigh. Therefore, the SGA concentrations of ecdysis (or in the exuviae) were qualitatively analyzed (Figure 2A). Neither α-chaconine nor α-solanine were detected in the foregut, because the foregut of insects is a small tube that does not digest and absorb food, and therefore food fragments are quickly transported into the midgut for subsequent digestion [44]. The midgut is the main site of nutrient absorption and sequestration [45]. In this study, the highest SGA contents being detected in the midgut could be attributed to the fact that the PTM samples were not starved before treatment. In addition, the accumulation content of α-chaconine in the PTM larvae was higher than that of α-solanine, possibly because α-chaconine is more toxic than α-solanine and takes a longer time to decompose in larvae [43]. Antibiotic-treated insect guts exhibited higher concentrations of SGA, while they had lower concentrations than those of the diet group. Further, the gut microbiota *Glutamicibacter halophytocola* S2 was recently proven to be related to SGA degradation [46].

None of the results of the present study suggested that antibiotic treatment had a direct effect on the larvae or even on the secondary metabolism products. However, Shen et al. [47] found that antibiotic treatment could decrease the weight and developmental duration of *Plutella xylostella* indirectly through the microbiome. In this study, we assumed that the antibiotics indirectly decreased SGA contents by affecting microorganisms. Ceja-Navarro et al. [35] demonstrated, using the defined diet supplemented with three broad-spectrum antibiotics (tetracycline, rifampicin and streptomycin), that gut microbiota played an important role in caffeine detoxification. At the same time, Lin et al. suggested that it was difficult for a single antibiotic to completely eliminate the gut bacteria of *Plutella xylostella* [48]. In this study, we used only two kinds of broad-spectrum antibiotics to control the gut bacteria of PTM larvae. The changes in the bacteria in the midgut after antibiotic treatment were not clear, and we could not be certain that the gut bacteria were completely controlled. The larvae treated with antibiotics and reinfected with SGAs recorded low α-chaconine and α-solanine concentrations, perhaps because the antibiotic treatment improved another way to decrease SGA contents. The changes of bacteria should be studied further.

Common insect detoxification strategies rely on degradation and excretion, while the mechanism of insect adaptation to plant secondary compounds is very complex [20,44,49]. Insects have evolved a number of strategies to tolerate plant toxins, such as the use of enzymes, cytochrome P450 genes and microbiomes, as well as some physiological strategies [50]. Insects possess specific enzymes to boost the bioactivity of toxins. Cytochrome P450 genes play important roles in the interactions of plants and insects. Specific transporters have been identified that allow insects to control the spatiotemporal dynamics of toxin accumulation [50,51]. These transporters work together and play important roles in the adaptation of insects to plant secondary metabolites. In this study, we explored the accumulation and excretion regulation of SGA in different tissues of potato tuber moth, as the methods used by PTM larvae use to defend against SGA derived from potato plants called for elucidation. Zhang et al. studied the genome assembly of *P. operculella* at the chromosomal level [52], which provided a significant resource for understanding the mechanism of PTM adaptation to SGA. However, further research on the adaptation mechanism of *P. operculella* is still needed.

## Figures and Tables

**Table 1:** Growth and development of potato tuber moth (PTM) larval instars fed on different varieties of potato leaves.

Cultivars	Larval Stage (d)	Instars	Larval Count	Head Capsule (mm)	Body Length (cm)	Body Weight (mg)
DS47	16.0 ± 0.62 ^a^	1	130	0.13 ± 0.08 ^bc^	0.14 ± 0.05 ^bc^	0.22 ± 0.04 ^a^
		2	90	0.27 ± 0.03 ^bc^	0.22 ± 0.03 ^bc^	1.05 ± 0.92 ^a^
		3	85	0.49 ± 0.11 ^b^	0.45 ± 0.16 ^a^	3.13 ± 0.90 ^a^
		4	60	0.90 ± 0.13 ^a^	0.98 ± 0.12 ^a^	8.10 ± 1.51 ^a^
QS9	12.0 ± 1.33 ^a^	1	110	0.17 ± 0.01 ^b^	0.16 ± 0.02 ^b^	0.18 ± 0.04 ^a^
		2	96	0.3 ± 0.01 ^b^	0.29 ± 0.04 ^b^	0.65 ± 0.36 ^b^
		3	80	0.49 ± 0.01 ^b^	0.46 ± 0.10 ^a^	1.54 ± 0.63 ^b^
		4	50	0.88 ± 0.09 ^ab^	0.95 ± 0.13 ^a^	4.48 ± 1.60 ^b^
LS6	14.5 ± 0.98 ^a^	1	127	0.29 ± 0.09 ^a^	0.28 ± 0.06 ^a^	0.18 ± 0.04 ^a^
		2	80	0.48 ± 0.08 ^a^	0.43 ± 0.05 ^a^	1.28 ± 1.17 ^a^
		3	60	0.69 ± 0.06 ^a^	0.66 ± 0.07 ^a^	3.63 ± 0.84 ^a^
		4	50	1.08 ± 0.07 ^a^	0.97 ± 0.07 ^a^	7.99 ± 1.50 ^a^
DS23	12.0 ± 1.02 ^a^	1	131	0.27 ± 0.06 ^a^	0.25 ± 0.04 ^a^	0.19 ± 0.03 ^a^
		2	90	0.45 ± 0.06 ^a^	0.37 ± 0.03 ^a^	0.65 ± 0.18 ^b^
		3	70	0.71 ± 0.0810 ^a^	0.58 ± 0.14 ^a^	2.49 ± 0.93 ^a^
		4	53	1.04 ± 0.1237 ^a^	0.92 ± 0.05 ^a^	7.26 ± 1.13 ^a^

Data are mean ± ΣEM. Different letters in the same columns indicate significant differences (*p* < 0.05, Tukey’s test).

**Table 2:** Reproduction parameters of PTM feeding on different potato varieties.

Cultivars	Pupal Stage	Pupal Weight (mg)	Pupation Rate	Emergence Rate	Fecundity (Eggs/Female)	Egg Stage (d)	Hatchability
DS47	8.0 ± 0.63 ^a^	9.47 ± 1.39 ^a^	84%	92%	89.5 ± 60.28 ^a^	5.63 ± 0.00 ^a^	76.7 ± 6.32 ^a^
QS9	6.0 ± 1.01 ^a^	7.07 ± 1.40 ^ab^	68%	86%	57 ± 36.35 ^b^	4.68 ± 0.04 ^a^	41 ± 3.11 ^b^
LS6	7.0 ± 0.33 ^a^	8.29 ± 1.09 ^a^	80%	90%	79.33 ± 51.68 ^a^	5.01 ± 0.01 ^a^	61.33 ± 5.13 ^ab^
DS23	5.5 ± 1.30 ^ab^	7.2 ± 1.22 ^a^	76.5%	80%	67.67 ± 44.12 ^a^	4.92 ± 0.06 ^a^	43.67 ± 2.08 ^b^

The means followed by different letters in the same columns are significantly different (*p* < 0.05, Tukey’s test).

**Table 3:** The mean (±SE) reproduction parameters of *P. operculella* for different SGA concentrations in the artificial diet.

Total SGA Concentration	Larval Time (d)	Pupal Weight (mg)	Pupation Rate	Emergence Rate	Sex Ratio	Fecundity	Hatchability
0 (ck)	7.2 ± 1.35 ^b^	5.23 ± 0.86 ^c^	41.67%	55.00%	6:5	35 ± 5.20 ^c^	15.01 ± 1.11 ^c^
0.1%	9.6 ± 2.21 ^b^	5.89 ± 0.95 ^bc^	53.36%	62%	8:5	42 ± 3.31 ^bc^	19.48 ± 0.90 ^c^
0.2%	14.3 ± 1.01 ^ab^	6.52 ± 0.65 ^a^	72.92%	90.63%	12:13	50 ± 4.36 ^b^	27.82 ± 0.37 ^bc^
0.3%	15.3 ± 0.92 ^a^	8.23 ± 1.42 ^a^	55.83%	95.45%	7:11	70 ± 10.21 ^a^	57.01 ± 3.39 ^a^
0.4%	15.8 ± 1.46 ^a^	6.8 ± 1.8 ^a^	40.21%	46.22%	3:7	29 ± 1.39 ^c^	8.79 ± 1.23 ^d^

The means followed by different letters in the same columns are significantly different (*p* < 0.05, Tukey’s test).

**Table 4:** Quantification using UHPLC–MS/MS of the role of bacteria in the transformation of SGA in PTM larvae.

Experiment	Treatment	α-Chaconine in PTM Larvae Midgut, Mean ± s.e (μg/g)	α-Solanine in PTM Larvae Midgut, Mean ± s.e (μg/g)
Control	PTM—normal microbiome	98.41 ± 1.22 ^a^	41.56 ± 1.12 ^a^
Antibiotic	PTM—antibiotic-treated	115.47 ± 6.53 ^b^	53.57 ± 1.93 ^a^
Antibiotic-reinfection	PTM—antibiotic-treated, reinfected with SGA	12.11 ± 1.08 ^c^	5.23 ± 0.06 ^b^

The means followed by different letters in the same columns are significantly different (*p* < 0.05, Tukey’s test).

## Data Availability

No new data were created or analyzed in this study. Data sharing is not applicable to this article.
